# Feasibility of a Specialized Large Language Model for Postgraduate Medical Examination Preparation: Single-Center Proof-Of-Concept Study

**DOI:** 10.2196/77580

**Published:** 2025-12-03

**Authors:** Yun Hao Leong, Lathiga Nambiar, Victoria Y J Tay, Sui An Lie, Ke Yuhe

**Affiliations:** 1 Division of Anesthesiology and Perioperative Medicine Singapore General Hospital Singapore Singapore; 2 Department of Anesthesiology Sengkang General Hospital Singapore Singapore

**Keywords:** large language model, artificial intelligence, technology acceptance model, medical education, postgraduate examination

## Abstract

**Background:**

Large language models (LLMs) are increasingly used in medical education for feedback and grading; yet their role in postgraduate examination preparation remains uncertain due to inconsistent grading, hallucinations, and user acceptance.

**Objective:**

This study evaluates the Personalized Anesthesia Study Support (PASS), a specialized GPT-4 model developed to assist candidates preparing for Singapore’s postgraduate specialist anesthesiology examination. We assessed user acceptance, grading interrater reliability, and hallucination detection rates to determine the feasibility of integrating specialized LLMs into high-stakes examination preparation.

**Methods:**

PASS was built on OpenAI’s GPT-4 and adapted with domain-specific prompts and references. Twenty-one senior anesthesiology residents completed a mock short answer question examination, which was independently graded by 3 human examiners and 3 PASS iterations. Participants reviewed feedback from both PASS and standard GPT-4 and completed a technology acceptance model (TAM) survey. Grading reliability was evaluated using Cohen and Fleiss κ. Hallucination rates were assessed by participants and examiners.

**Results:**

Of the 21 participants, 17 (81%) completed the TAM survey, generating 136 responses. PASS scored significantly higher than standard GPT-4 in usefulness (mean 4.25, SD 0.50 vs mean 3.44, SD 0.82; *P*<.001), efficiency (mean 4.12, SD 0.61 vs mean 3.41, SD 0.74; *P*<.001), and likelihood of future use (mean 4.13, SD 0.75 vs mean 3.59, SD 0.90; *P*<.001), with no significant difference in ease of use (mean 4.56, SD 0.63 vs mean 4.50, SD 0.61; *P*=.35). Internal grading reliability was moderate for PASS (κ=0.522) and fair for human examiners (κ=0.275). Across 316 PASS-generated responses, 67 hallucinations and 189 deviations were labeled. Hallucination labeling rates were comparable between candidates (10/67, 15%) and examiners (57/249, 22.9%; *P*=.21), while examiners labeled significantly more deviations (168/249, 67.5% vs 21/67, 31%; *P*<.001).

**Conclusions:**

PASS demonstrated strong user acceptance and grading reliability, suggesting feasibility in high-stakes examination preparation. Experienced learners could identify major hallucinations at comparable rates to examiners, suggesting potential in self-directed learning but with continued need for caution. Further research should refine grading accuracy and explore multicenter evaluation of specialized LLMs for postgraduate medical education.

## Introduction

The integration of artificial intelligence (AI) into education has accelerated in recent years, particularly with the rapid evolution of large language models (LLMs) such as OpenAI’s GPT-4 and Anthropic’s Claude. These models employ transformer-based deep learning architectures to process and generate human-like text, enabling dynamic adaptation across educational domains. In medical education, LLMs have been applied for automated question generation, simulated case discussions, feedback provision, and adaptive tutoring [1]. Recent reviews highlight that LLMs can support formative learning through contextual explanations and feedback personalization while also noting persistent issues in transparency, bias, and factual accuracy [2-4].

Despite these promising uses, major challenges remain in applying LLMs to high-stakes assessment. Studies on LLM-based grading report inconsistent results, including conservative grading patterns, variable interrater reliability (IRR) with human assessors [5], and context-dependent scoring variability [6]. Even advanced LLMs such as GPT-4 have shown inconsistent scoring in complex clinical assessments, underscoring the importance of standardized rubrics, transparent prompt reporting, and model calibration [7,8]. Most prior work has evaluated general-purpose LLMs with minimal adaptation, which limits their accuracy in assessing domain-specific medical content. While prompt engineering has been explored to optimize response consistency [9], few studies have explored the feasibility of specialized LLMs tailored to specific educational needs.

Another major concern surrounding LLMs in education is the phenomenon of hallucinations, where models generate responses that appear plausible but are factually incorrect or misleading [10]. Previous studies have reported hallucination rates as high as 27.1% in LLM-generated feedback [11], raising concerns about the potential for misinformation. Recent work in medical education has categorized hallucinations by cognitive level and clinical severity, noting that even subtle deviations can distort learners’ conceptual understanding [12]. Given the high-stakes nature of medical training, it is essential to understand not only how frequently these hallucinations occur but also the extent to which educators and students can detect and mitigate their impact.

In parallel, user acceptance strongly influences whether LLM-based systems are adopted into medical curricula. Although several studies report favorable perceptions of LLM-assisted educational tools among undergraduate students [13], there remains a gap in understanding postgraduate learners’ perceptions, particularly in specialist examination preparation. Regardless of technical performance, effective integration of LLMs into educational practice depends on user acceptance, which is shaped by perceived usefulness, ease of use, and trustworthiness [14].

To address these issues, our study aims to develop and pilot the Personalized Anesthesia Study Support (PASS), a specialized GPT designed to provide grading and feedback for candidates preparing for Singapore’s postgraduate specialist examination in anesthesiology. We present this as a proof-of-concept study to explore the feasibility and potential role of specialized LLMs as supplemental tools in postgraduate examination preparation. Our primary objective was to assess end user acceptance of PASS by using a technology acceptance model (TAM) survey. Secondary objectives included evaluating the grading reliability compared to human examiners and analyzing the hallucination detection trends.

## Methods

### Ethical Considerations

This study was conducted at Singapore General Hospital under the SingHealth Anesthesiology Residency Program. It was reviewed by the Singapore General Hospital Research Office and was granted exemption from formal institutional review board review, as it involved routine educational activities and the use of anonymized resident data. Participation was voluntary, and no identifiable personal data were collected. Completion of the TAM survey was considered implied consent. All data were deidentified prior to analysis, and no compensation was provided to the participants.

### Recruitment and Study Participants

Participants were recruited in a systematic cohort-based approach within the SingHealth Anesthesiology Residency Program at Singapore General Hospital. All anesthesiology residents actively preparing for an upcoming Master of Medicine (MMed) anesthesiology examination were scheduled to complete a routine, in-person mock short answer question (SAQ) assessment and were invited to participate in the study. Eligibility required active enrollment in the residency program with at least 32 months of anesthesia-related training experience, consistent with the criteria for sitting in the MMed examination. No additional exclusion criteria were applied, as the cohort was already homogeneous in the training level and examination eligibility. At the start of the session, a member of the program faculty provided a standardized study briefing, explaining its objectives, procedures, and voluntary nature of participation. Residents were informed that participation would not influence their training evaluation or examination outcomes. Implied consent was obtained following this briefing. A total of 21 residents, representing the full cohort of candidates preparing for that year’s MMed examination, consented and participated in the study. The cohort comprised residents in years 2 to 4 of training, with a gender distribution of 14 females and 7 males.

### Development Process of PASS

PASS was developed using OpenAI’s GPT-4 model and customized via the ChatGPT Custom GPT interface to provide grading and detailed feedback for SAQ-style responses. Two study investigators (LYH and KY) conducted 3 iterative rounds of pilot testing by using simulated essay responses modeled after previous MMed anesthesiology examination questions. Each round involved a systematic review of PASS outputs by both investigators and 2 faculty anesthesiologists to evaluate the clinical accuracy, feedback clarity, and grading consistency. Refinements across iterations focused on clarifying role-based prompts, improving alignment with examiner expectations, and reducing hallucinated or ambiguous statements in the generated feedback. After each testing round, the prompt was revised to enhance the specificity of the instructions and the standardization of the grading language. The final prompt version, presented in Table S1 in Multimedia Appendix 1, reflected cumulative adjustments derived from these pilot evaluations and represented the configuration used in the study.

The reference materials in PASS included the 2024 MMed anesthesiology syllabus (PDF, 32 pages), examiner feedback reports (n=5, 1-2 pages each), past SAQ papers (2018-2024), and standard textbooks such as Miller’s Anesthesia (9th edition) and Yao & Artusio’s Anesthesiology: Problem-Oriented Patient Management (9th edition).

Prompt engineering followed the principles outlined by Meskó [15], focusing on specificity and contextualization. Role-playing prompts were used to instruct the LLM to adopt the perspective of an anesthesiologist examiner, ensuring clinically accurate and assessment-calibrated evaluations.

### Study Design and Grading Evaluation

Participants completed a closed-book mock examination of 4 SAQs. These were adapted from prior internal mock examinations aligned with recent MMed anesthesiology examination themes and reviewed by another anesthesiology faculty to ensure content validity. Examiner 1 developed a standardized mark scheme and graded all 84 scripts as per standard assessment practices. Each SAQ constituted 1 script, with 21 participants producing 84 total responses.

Additionally, 2 independent human examiners (examiners 2 and 3) also graded all the scripts based on the standardized mark scheme. Examiners 2 and 3 independently evaluated the PASS grading iterations alongside examiner 1. Two study investigators generated 3 separate PASS grading iterations per script in parallel chat windows. PASS was not provided with the official grading rubric and relied solely on its internal knowledge base. SAQ responses were graded on a 0-8 scale and stratified into poor (0-4.5), average (5-5.5), and good (6-8) categories. The stratified gradings were then analyzed for grading IRR, with examiner 1’s scores serving as the reference standard when required.

### TAM Evaluation

Participants subsequently evaluated both PASS and standard GPT-4.0 feedback by using a customized TAM survey [16]. TAM is grounded in the theory of reasoned action, which posits that an individual’s behavioral intention to use a technology is shaped by their attitudes toward the technology and by perceived usefulness. This framework has been widely applied to understand user acceptance of new technologies, including those used in medical education [14,17,18]. Four dimensions were rated on a 5-point Likert scale (1=least agreement, 5=strongest agreement): (1) usefulness for examination preparation, (2) efficiency in study preparation, (3) ease of use, and (4) likelihood of future use. Participation in the survey was voluntary and anonymous. Incomplete surveys were excluded from the analysis.

### Hallucination and Deviation Analysis

PASS responses and feedback to participant essays were reviewed independently by study investigators and participants to detect hallucinations and deviations. Hallucinations were defined as “any incorrect information or statements that may cause moderate to major patient harm,” while deviations were defined as “variations from usual clinical practice unlikely to result in patient harm.” The frequency of hallucinations and deviations detection was compared between groups.

### Statistical Analysis

All statistical evaluations were performed in the Excel and Python 3.8 environment. The TAM scores for PASS versus GPT-4 were compared using paired 2-sided *t* tests. Fleiss κ was used to calculate IRR within PASS and human examiner groups, and agreement between individual examiners was evaluated using Cohen κ analysis. Chi-square tests were used to compare hallucination and deviation detection between examiners and candidates. As this was a proof-of-concept feasibility study, no a priori power calculation was performed.

This paper adheres to the STROBE (Strengthening the Reporting of Observational Studies in Epidemiology) reporting guideline [19], and a completed STROBE checklist has been included in Multimedia Appendix 2.

## Results

### TAM Results

**Figure 1:**
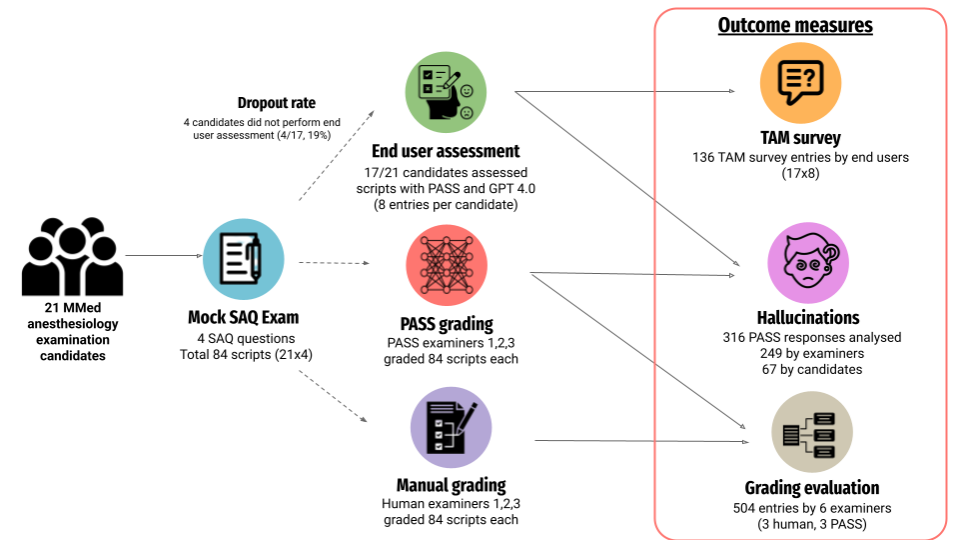
Flow diagram summarizing the design and data pathways of this study. A total of 21 MMed anesthesiology residents participated in a mock SAQ examination comprising 4 questions (84 total scripts). Each script was independently graded by 3 human examiners and 3 iterations of the specialized large language model (PASS 1-3). MMed: Master of Medicine; PASS: Personalized Anesthesia Study Support; SAQ: short answer question; TAM: technology acceptance model.

Of the 21 participants, 17 (81%) completed the TAM survey, giving a total of 136 survey responses. Four participants did not complete the TAM survey, and no data imputation was performed. User perceptions of PASS were positive. Mean ratings for PASS were as follows: usefulness (4.25, SD 0.50), efficiency (4.12, SD 0.61), ease of use (4.56, SD 0.63), and likelihood of future use (4.13, SD 0.75). Paired 2-sided *t* tests comparing PASS to standard GPT-4 revealed statistically significant differences in 3 of the 4 domains. PASS was rated significantly higher than GPT-4 in usefulness (mean difference=0.81, 95% CI 0.66-0.96; *P*<.001), efficiency (mean difference=0.71, 95% CI 0.53-0.88; *P*<.001), and likelihood of future use (mean difference=0.54, 95% CI 0.39-0.70; *P*<.001). There was no significant difference between PASS and GPT-4 in ease of use (mean difference=0.06, 95% CI –0.07 to 0.18; *P*=.35). A summary of the TAM results can be found in Table 1 and Figure 1.

**Table 1:** Technology acceptance model survey results comparing PASSa and standard GPT-4 in a single-center observational feasibility study involving postgraduate anesthesiology residents (N=21).

Question	PASS, mean (SD)	GPT, mean (SD)	Mean difference (95% CI)	*P* value
Useful for my examination preparation	4.25 (0.5)	3.441 (0.817)	0.809 (0.66 to 0.96)	<.001
Helps me to study efficiently	4.118 (0.612)	3.412 (0.738)	0.706 (0.53 to 0.88)	<.001
Easy to use	4.559 (0.632)	4.5 (0.611)	0.059 (–0.07 to 0.18)	.35
I will use it in future examinations	4.132 (0.751)	3.588 (0.902)	0.544 (0.39 to 0.70)	<.001

^a^PASS: Personalized Anesthesia Study Support.

### Grading Evaluation

**Figure 2:**
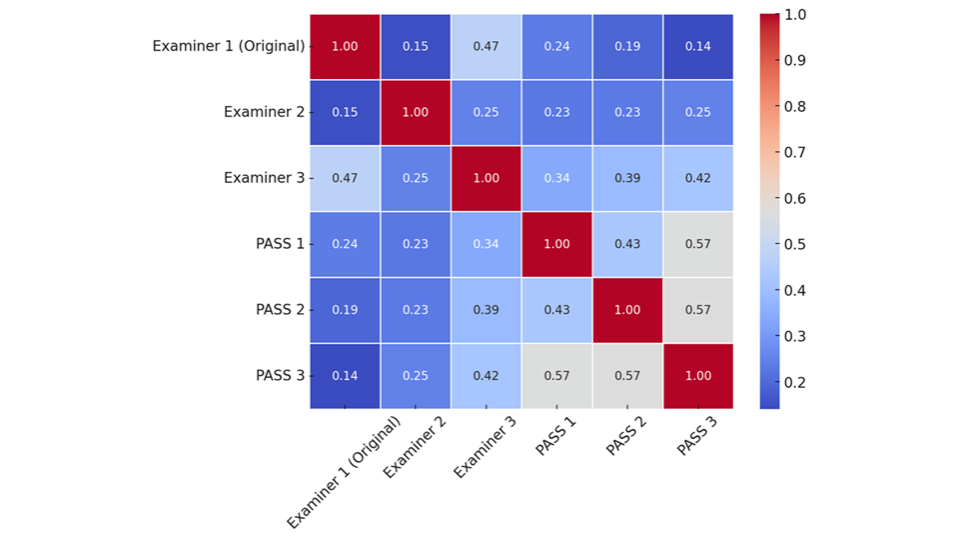
Interexaminer agreement matrix heatmap showing pairwise Cohen κ values differing among human examiners (examiners 1-3) and PASS iterations (PASS 1-3). Each cell represents the level of agreement between 2 graders, with values ranging from 0 (no agreement) to 1 (perfect agreement). Darker red shading indicates higher agreement, lighter blue tones represent moderate agreement, and deep blue tones indicate lower agreement. PASS iterations demonstrated higher internal consistency (κ=0.52-0.57) compared with human examiners (κ=0.15-0.47), suggesting more standardized grading performance across large language model assessments. PASS: Personalized Anesthesia Study Support.

A total of 504 scripts were marked by 6 examiners (3 humans, 3 PASS). PASS examiners demonstrated moderate internal IRR (κ=0.522), whereas human examiners exhibited fair internal IRR (κ=0.275), based on the benchmarks of Landis and Koch [20]. Individually, the agreement of PASS and human examiners with examiner 1 were as follows: PASS 1 (κ=0.237), PASS 2 (κ=0.193), PASS 3 (κ=0.140), examiner 2 (κ=0.149), and examiner 3 (κ=0.470). Among these, examiner 3 demonstrated the highest agreement with examiner 1, while PASS 3 exhibited the lowest. The complete interexaminer agreement matrix can be found in Figure 2. When analyzed as a group (Table 2), PASS examiners approached a moderate level of agreement with the combined group of human examiners (examiners 1, 2, and 3) (κ=0.357, 95% CI 0.156-0.558). Pairwise agreement between PASS and individual human examiners ranged from κ=0.159 (PASS vs examiner 1, slight) to κ=0.429 (PASS vs examiner 3, moderate).

**Table 2:** Interexaminer agreement between PASSa and human examiners in a single-center observational feasibility study among anesthesiology residents (N=21).

Comparison	κ score (95% CI)
PASS versus combined examiners (1,2,3)	0.357 (0.156 to 0.558)
PASS versus examiner 1	0.159 (–0.053 to 0.372)
PASS versus examiner 2	0.193 (–0.017 to 0.404)
PASS versus examiner 3	0.429 (0.228 to 0.630)

^a^PASS: Personalized Anesthesia Study Support.

### Hallucination and Deviation Detection Rates

A total of 316 PASS-generated responses were evaluated for hallucinations and deviations. Examiners conducted 78.8% (249/316) of the evaluations, while candidates accounted for 21.2% (67/316). Across all responses, 67 hallucinations and 189 deviations were labeled. Each hallucination or deviation was labeled by independent reviewers. No statistically significant difference was observed in the hallucination detection rates between candidates and examiners (*P*=.21). Candidates identified 10 hallucinations across 67 responses (15%), while examiners detected 57 hallucinations across 249 responses (22.9%). In contrast, examiners identified significantly more deviations than candidates. Candidates detected 21 deviations (31.3%), whereas examiners identified 168 deviations (67.5%)—a difference that was highly significant (*P*<.001) (Table 3).

**Table 3:** Comparison of hallucination and deviation labeling by candidates and examiners. Values indicate detection rate (%). Differences were tested using chi-square analysis.

	Candidates (n=67), n (%)	Examiners (n=249), n (%)	*P* value
Hallucinations identified	10 (14.9)	57 (22.9)	.21
Deviations identified	21 (31.3)	168 (67.5)	<.001

## Discussion

### Primary Outcome: Technology Acceptance and User Perceptions

This proof-of-concept study demonstrates strong end user acceptance of a specialized LLM, supporting the potential role of tailored AI tools in high-stakes medical examination preparation. Younger learners increasingly prefer technology-driven educational tools that provide prompt feedback, personalized learning experiences, and interactive engagement [21,22]. Specialized LLM platforms such as our case example PASS offers an innovative approach to meet these expectations.

Our findings are consistent with prior research showing that LLM-based educational tools are generally well-received across educational contexts [14,23,24]. However, user acceptance specifically for high-stakes examination preparation, where performance directly impacts career progression, remains underexplored [25]. Given that user acceptance is a key factor in the successful adoption of new technologies [26], our results add evidence that specialized LLMs can be successfully incorporated as study aids for postgraduate medical examinations.

Notably, PASS, a specialized version of GPT-4 customized with domain-specific content, received significantly higher ratings in usefulness, efficiency, and likelihood of future use compared to standard GPT-4. This supports the argument that domain-specialized enhancements improve both learner experience and perceived educational value [27].

### Grading Reliability

In our study, PASS demonstrated a higher internal grading IRR (κ=0.522) compared to human examiners (κ=0.275), suggesting that LLM-based grading may provide more standardized evaluations. When analyzed as a group, PASS examiners showed statistically significant agreement with human examiners (κ=0.357), indicating that PASS grading trends align with human assessment.

Timely and accurate grading feedback is crucial for effective examination preparation, as it helps candidates refine their responses and align their answering techniques with examiner expectations [28]. However, faculty availability is often limited, restricting or delaying access to grading opportunities. Additionally, human grading is inherently variable, influenced by factors such as individual biases and grading fatigue [29]. Our results suggest that specialized LLMs could offer scalable, consistent supplemental grading and feedback in postgraduate education, potentially alleviating faculty workload and enabling self-directed formative assessment.

However, PASS’s grading agreement with humans remained only moderate, indicating that its reliability may not yet be sufficient for formal grading. This finding aligns with previous research highlighting the limitations of LLMs as formal grading tools [30]. Despite these constraints, specialized LLMs remain valuable as self-assessment tools, allowing students to estimate their performance during examination preparation.

PASS was intentionally not provided with the official marking rubric to reflect the realistic conditions of learner-driven use. This design choice likely contributed to moderate agreement levels, as rubric-guided calibration could enhance consistency between AI and human grading. Future studies may investigate whether standardized grading frameworks improve IRR and alignment with human examiners.

### Hallucinations and Deviations in AI-Generated Feedback

Hallucinations in LLM generated feedback are a well-documented concern [1]. In medical education, this issue is particularly critical, as even minor misconceptions can lead to significant errors in clinical management, potentially resulting in adverse patient outcomes [31]. While previous studies have discussed potential ways to mitigate the effect of hallucinations [32], there is a notable gap in research comparing the detection rate of hallucinations between tutors and learners.

Encouragingly, our study shows that candidates were able to identify hallucinations at a rate comparable to examiners. The ability of candidates to recognize and filter out major erroneous feedback on their own suggests that specialized LLMs show potential for use under supervision in examination preparation. However, it is important to note that all candidates in our study had at least 32 months of anesthesia experience, which may have contributed to their ability to detect errors effectively. Less experienced learners may not demonstrate the same level of discernment and could require closer supervision when using AI-generated feedback.

Additionally, our results show that candidates were significantly less likely than examiners to detect deviations from standard clinical practice. Failure to recognize clinically relevant, even if minor, deviations from standard practice may inadvertently reinforce incorrect reasoning patterns. If undetected, such deviations could perpetuate unsafe habits or incomplete understanding, which, in a clinical context, might affect patient management. Incorporating structured faculty review or critical appraisal exercises into curricula could help learners identify and mitigate such errors when using AI feedback.

Our findings suggest important considerations for future use of LLMs in education. Educators must assess whether learners possess the critical skills to evaluate AI-generated content before incorporating LLM feedback into curricula. Future research should explore hallucination detection across learner levels and strategies to build critical evaluation skills alongside AI-assisted learning.

### Limitations and Future Work

This study has several limitations. First, the small, single-center cohort of 21 participants may limit the generalizability of our findings. All participants were experienced anesthesiology residents from the same training program and were approaching their final examinations. Consequently, the results may not extend to less experienced trainees, learners from other specialties, or different educational settings. Junior trainees may have greater difficulty discerning hallucinations or subtle deviations in AI-generated feedback, underscoring the continued importance of supervised use and faculty guidance. Future multicenter studies involving a broader range of specialties, experience levels, and training environments will be essential to evaluate the scalability and educational impact of specialized LLMs. Second, no formal power calculation was conducted, given the exploratory, proof-of-concept nature of the study. In addition, intraexaminer reliability was not assessed, which may have influenced the variability in human grading. Finally, this study did not examine the direct effect of using PASS on actual examination performance or learning outcomes. Assessing these educational and behavioral impacts should be a key focus of subsequent research to determine the long-term value and safety of integrating specialized LLMs into postgraduate medical education.

Building on these preliminary findings, future research should focus on multicenter validation of specialized LLMs across different institutions, specialties, and learner experience levels to better define their generalizability. Further work is also needed to evaluate the longitudinal educational impact of LLM-assisted feedback on examination performance, critical thinking, and clinical reasoning. Incorporating standardized grading rubrics or structured prompt frameworks may improve grading reliability and enhance alignment with human examiners. In parallel, research should explore strategies to train learners in AI literacy and critical appraisal, ensuring they can appropriately interpret and verify model outputs. Ultimately, integrating specialized LLMs into postgraduate education should aim to augment, not replace, human judgment, fostering safe, reflective, and supervised use of AI in medical learning environments.

### Conclusions

This study provides proof-of-concept evidence supporting the feasibility and user acceptance of specialized LLMs as supplemental study aids for postgraduate medical examination preparation. The specialized LLM (PASS) demonstrated strong user acceptance, outperforming standard GPT-4 in perceived usefulness, study efficiency, and likelihood of future use. PASS also showed moderate grading consistency and agreement with human examiners, indicating potential value in supporting formative, self-directed learning.

While experienced learners were able to recognize major hallucinations at rates comparable to examiners, their lower detection of subtle deviations underscores the continued necessity of human oversight when using AI-generated educational feedback. These findings highlight the potential of specialized LLMs to enhance postgraduate learning under supervision while reinforcing the importance of ongoing faculty involvement, quality control, and iterative model refinement before broader integration into training programs.

## Appendix

Multimedia Appendix 1 Personalized Anesthesia Study Support prompt.

Multimedia Appendix 2 STROBE checklist.

## Abbreviations

AI: artificial intelligence
IRR: interrater reliability
LLM: large language model
MMed: Master of Medicine
PASS: Personalized Anesthesia Study Support
SAQ: short answer question
STROBE: Strengthening the Reporting of Observational Studies in Epidemiology
TAM: technology acceptance model

## Data Availability

The datasets generated and analyzed during this study are not publicly available due to its sensitive nature but are available from the corresponding author on reasonable request. Deidentified data supporting the findings of this study may be shared for academic or research purposes upon submission of an appropriate data use agreement.
